# B Vitamins, Methionine and Alcohol Intake and Risk of Colon Cancer in Relation to *BRAF* Mutation and CpG Island Methylator Phenotype (CIMP)

**DOI:** 10.1371/journal.pone.0021102

**Published:** 2011-06-27

**Authors:** Eva S. Schernhammer, Edward Giovannucci, Yoshifumi Baba, Charles S. Fuchs, Shuji Ogino

**Affiliations:** 1 Channing Laboratory, Department of Medicine, Brigham and Women's Hospital and Harvard Medical School, Boston, Massachusetts, United States of America; 2 Ludwig Boltzmann-Institute for Applied Cancer Research, KFJ-Spital, Vienna, Austria; 3 Applied Cancer Research - Institute for Translational Research Vienna (ACR–ITR VIEnna), Vienna, Austria; 4 Department of Epidemiology, Harvard School of Public Health, Boston, Massachusetts, United States of America; 5 Department of Nutrition, Harvard School of Public Health, Boston, Massachusetts, United States of America; 6 Department of Medical Oncology, Dana-Farber Cancer Institute, Boston, Massachusetts, United States of America; 7 Department of Pathology, Brigham and Women's Hospital and Harvard Medical School, Boston, Massachusetts, United States of America; The Chinese University of Hong Kong, Hong Kong

## Abstract

**Background:**

One-carbon metabolism appears to play an important role in DNA methylation reaction. Evidence suggests that a low intake of B vitamins or high alcohol consumption increases colorectal cancer risk. How one-carbon nutrients affect the CpG island methylator phenotype (CIMP) or *BRAF* mutation status in colon cancer remains uncertain.

**Methods:**

Utilizing incident colon cancers in a large prospective cohort of women (the Nurses' Health Study), we determined *BRAF* status (N = 386) and CIMP status (N = 375) by 8 CIMP-specific markers [*CACNA1G*, *CDKN2A* (p16), *CRABP1*, *IGF2*, *MLH1*, *NEUROG1*, *RUNX3*, and *SOCS1*], and 8 other CpG islands (*CHFR*, *HIC1*, *IGFBP3*, *MGMT*, MINT-1, MINT-31, p14, and *WRN*). We examined the relationship between intake of one-carbon nutrients and alcohol and colon cancer risk, by *BRAF* mutation or CIMP status.

**Results:**

Higher folate intake was associated with a trend towards low risk of CIMP-low/0 tumors [total folate intake ≥400 µg/day vs. <200 µg/day; the multivariate relative risk = 0.73; 95% CI = 0.53–1.02], whereas total folate intake had no influence on CIMP-high tumor risks (P_heterogeneity_ = 0.73). Neither vitamin B_6_, methionine or alcohol intake appeared to differentially influence risks for CIMP-high and CIMP-low/0 tumors. Using the 16-marker CIMP panel did not substantially alter our results. B vitamins, methionine or alcohol intake did not affect colon cancer risk differentially by *BRAF* status.

**Conclusions:**

This molecular pathological epidemiology study suggests that low level intake of folate may be associated with an increased risk of CIMP-low/0 colon tumors, but not that of CIMP-high tumors. However, the difference between CIMP-high and CIMP-low/0 cancer risks was not statistically significant, and additional studies are necessary to confirm these observations.

## Introduction

DNA methylation is an important epigenetic mechanism in gene silencing and imprinting [1], [2], [3]. Aberrant epigenetic silencing of tumor suppressors by CpG island hypermethylation is commonly observed in human malignancies. The CpG island methylator phenotype (CIMP) is characterized by propensity for widespread CpG island hypermethylation [4], [5]. High degree of CIMP (CIMP-high) is a distinct phenotype in colorectal cancer, associated with older age, female gender, proximal tumor location, microsatellite instability, *BRAF* mutation, and high-level tumor LINE-1 methylation [6], [7], [8], [9]. However, etiologic factors for CIMP-high remain largely speculative. Despite a strong association between CIMP and *BRAF* mutation in colorectal cancer [5], [6], [7], [8], the hypothesis that *BRAF* mutation causes aberrant CpG island methylation [10] has been highly controversial [11]. Recently, DNMT3B has emerged as one of causes of CpG island methylation in tumor [12], [13], [14]; nonetheless, the association between DNMT3B and CIMP in colorectal cancer is at most modest [13] and tumor CpG island methylation appears to be influenced by additional factors.

Folic acid and related vitamins B_2_, B_6_ and B_12_, are essential for DNA methylation and the production of purine and pyrimidine nucleotides required for DNA synthesis. Considerable epidemiological evidence suggests that a low-folate diet is associated with an increased risk of colorectal cancer [15], [16], [17]. Likewise, alcohol consumption increases colorectal cancer risk [18], likely through its anti-folate effects [19]. We have recently shown that adequate folate intake and low consumption of alcohol are protective against LINE-1 hypomethylated colon cancer, but not against LINE-1 hypermethylated colon cancer [20]. While a recent study demonstrated an association between folate and CpG island methylation in normal colorectal tissue [21], how folate or other B vitamin intake influences CpG island methylation or *BRAF* mutation in tumor remains less well understood, and the literature data are somewhat limited [22], [23], [24], [25].

We therefore assessed whether the influence of folate on colon cancer risk differed according to CIMP and *BRAF* status. For this purpose, we used tumor specimens from a prospective cohort study that has previously shown that folate intake was inversely associated with the risk of colon cancer [26]. The availability of detailed and updated information on folate intake and tumor specimens within this cohort permitted a more comprehensive examination of the effect of folate intake on risk of developing CIMP-high and non-CIMP-high colon cancers as well as by *BRAF* mutational status. Specificity in the association between folate and colon cancer to particular tumor phenotypes would further enhance the case for causality and would provide important insights into the carcinogenic mechanisms of folate deficiency.

## Materials and Methods

### Study Subjects

The Nurses' Health Study (NHS) was established in 1976 when 121,701 U.S. female registered nurses, 30 to 55 years of age, completed a mailed questionnaire [27]. Follow-up within the cohort currently exceeds 92%. We mailed biennial questionnaires to update information and identify newly diagnosed cases of cancer. In 1980, the NHS questionnaire was expanded to include a validated assessment of diet, and updated dietary assessments have been conducted every four years [28]. The institutional review boards at Brigham and Women's Hospital and the Harvard School of Public Health approved this study and the consent procedure.

### Assessment of Nutrients

Dietary intake of various nutrients including folate, vitamin B_6_, B_12_, and methionine were assessed by self-administered semiquantitative food frequency questionnaires (SFFQ), which were completed in 1980, 1984, 1986, 1990, 1994, and 1998. Nutrient intakes were calculated by multiplying the reported frequency of consumption of each specified food item by the nutrient content of the specified portion size and then summing these products for all food items. Information on multivitamin use and the use of other supplements was also collected, including details on which brand name and type. An extensive database of supplement formulation was then used to calculate specific nutrient contributions from these supplemental sources. These nutrient contributions were subsequently added to the specific nutrient intake from foods to calculate a daily intake for each woman. This method of dietary assessment has been extensively validated and its reliability evaluated [28]. The correlation coefficient was 0.55 for the correlation between total folate intake calculated from the 1980 questionnaire and erythrocyte folate concentrations measured in 1987 in this cohort [29]. Moreover, vitamin B_6_ intake as assessed by 1980, 1984, and 1986 SFFQs has been shown to correlate with one-week diet records, with correlations ranging from 0.54 to 0.58 [28], [30].

### Assessment of Other Covariates

Alcohol consumption was the sum of the values for three types of beverages: beer, wine, and spirits. We assumed an ethanol content of 13.1 g for a 12-ounce (38-dl) can or bottle of beer, 11.0 g for a 4-ounce (12-dl) glass of wine, and 14.0 g for a standard portion of spirits. In validation studies, the correlation coefficient for the correlation between alcohol consumption derived from the 1980 SFFQ and the average of four one-week diet records was 0.90 [31]. Other risk factors for colon cancer such as physical exercise and body mass index have generally been assessed biennially on the main questionnaires.

### Ascertainment of Colon Cancer Cases and Tumor Tissue Procurement

We included colon cancers reported on the biennial questionnaires between the return of the 1980 questionnaire and June 1, 2004. With permission from study participants, colon cancer was confirmed through physicians' review of the nurses' medical records. If permission was denied, we attempted to confirm the self-reported cancer with an additional letter or phone call. We also searched the National Death Index to identify deaths among the nonrespondents to each two-year questionnaire. The computerized National Death Index is a highly sensitive method for identifying deaths in this cohort [32]. For all deaths attributable to colon cancer, we requested permission from family members (subject to state regulation) to review the medical records. We successfully obtained specimens for 58% of cases (n = 527) over 22 years of follow-up in NHS. There was no significant difference in demographic or exposure variables between cases with available tissue and those without available tissue [33].

### Genomic DNA extraction and sequencing of *BRAF*

Genomic DNA from paraffin-embedded tissue was extracted, and whole genome amplification was performed by PCR using random 15-mer primers [34]. PCR and sequencing targeted for *BRAF* codon 600 were performed as previously described [35].

### Real-time PCR (MethyLight) for quantitative DNA methylation

To determine CIMP status, we quantified DNA methylation in eight CIMP-specific promoters. Bisulfite treatment on genomic DNA and subsequent real-time PCR (MethyLight) [36] were validated and performed as previously described [37]. The eight CIMP-specific promotors that we quantified (*CACNA1G*, *CDKN2A* (p16), *CRABP1*, *IGF2*, *MLH1*, *NEUROG1*, *RUNX3*, and *SOCS1*) [5], [38] were selected from screening of 195 CpG islands [5], [39]. CIMP-high was defined as ≥6/8 methylated markers using the eight-marker CIMP panel, and CIMP-low/0 was defined as 0/8 to 5/8 methylated markers, according to the previously established criteria [38]. In secondary analyses, we added eight more markers (*CHFR*, *MGMT*, *P14*, *WRN*, *HTC1*, *MINT1*, *MINT31*, *IGFBP3*) [7] for a 16-marker panel. Using this 16-marker panel, we created two categories of CIMP (CIMP-high defined as ≥11/16 methylated markers; and CIMP-low/0 as 0/16 to 10/16 methylated markers). Concordance of CIMP-high diagnosis between the 8-marker and 16-marker panels was high (0.97; k = 0.89) [7].

### Statistical Analysis

We excluded women who did not complete the baseline 1980 dietary questionnaire or recorded implausible dietary data (n = 29,279), reported a baseline history of cancer (except non-melanoma skin cancer; n = 3,627), inflammatory bowel disease, hereditary nonpolyposis colon cancer, or a familial polyposis syndrome (n = 103), or had died prior to baseline (n = 1). After these exclusions, 88,691 women were eligible for analysis and accrued follow-up time beginning on the month of return of their baseline questionnaire and ending on the month of diagnosis of colon cancer, death from other causes, or June 1, 2002, whichever came first. In a previous analysis of this cohort, folate intake was significantly associated with the risk of colon cancer but had no influence on the risk of rectal cancer [26]; as a result, we did not consider incident rectal cancer among the study participants in this analysis. Like rectal cancer cases, cases of colon cancer for which we were unable to quantified DNA methylation were censored from the analyses at their date of diagnosis and were not included as endpoints.

We calculated incidence rates of colon cancer for participants in a specific category of folate intake by dividing the number of incident cases by the number of person-years. We computed relative risks (RR) by dividing the incidence rate in one category by the incidence rate in the reference category and used Cox proportional hazards modeling to control for multiple variables simultaneously and to compute 95% confidence intervals (CI). With the exception of folate, vitamin B_6_, methionine, and alcohol, for which we used baseline information in our primary analyses, we used the most updated information for all covariates prior to each two-year interval.

To compare the specific effect of intake of folate and other nutrients on colon cancer risk according to CIMP and *BRAF* status, we employed a previously described method of competing risk analysis utilizing duplication method Cox regression [40], [41]. This methodology permits estimation of separate regression coefficients for nutrient intake stratified by the type of outcome [e.g. CIMP-high cancer vs. CIMP-low/0 cancer; *BRAF* mutation (+) vs. (−)]. We assessed the statistical significance of the interaction between the risk estimates according to tumor type using a likelihood ratio test that compared the model that allowed for separate associations of folate and other nutrients according to CIMP/*BRAF* status with a model that assumed a common association. We conservatively interpreted statistical significance of findings because of multiple hypothesis testing. To take into account multiple testing (five one-carbon nutrients were examined) we adjusted our significance level to p = 0.01 ( = 0.05/5). We used SAS version 9.1.3 (Cary, NC) for all analyses. All P values are two-sided.

## Results

Among 88,691 women included in these analyses, those with a baseline folate intake of less than 200 µg/day were slightly more likely to smoke and to be sedentary, compared to women with 400 µg daily folate intake or more (Table 1). They were also less likely to use aspirin or postmenopausal hormones regularly. In addition, only 8% of women with less than 200 µg/day folate intake used multivitamins, whereas among those with 400 µg folate or more daily, 84% reported current multivitamin use. Further details have been reported elsewhere [42].

**Table 1:** Baseline characteristics of the Nurses' Health Study cohort*.

	Energy-adjusted Folate Intake, µg/day			
	<200	200–299	300–399	≥400
**Characteristic** *	N = 20,907	N = 28,882	N = 12,997	N = 25,905
**Dietary Intake** Ψ				
Folate (µg/day)	159	246	341	677
Vitamin B_6_ (mg/day)	1.59	2.05	2.76	2.15
Vitamin B_12_ (mg/day)	5.55	6.45	7.78	15.06
Alcohol (g/day)	6.35	6.37	6.00	6.33
Methionine (mg/day)	1.74	1.86	1.95	1.93
Calcium (mg/day)	607	723	798	812
Beef, pork, or lamb as a main dish (servings per week)	0.44	0.37	0.32	0.33
**Other Characteristics** *				
Median age (yr)	46.6	46.8	46.8	46.6
Former or current smoker (%)	60	56	54	55
Pack-years†	23.3	20.4	18.7	19.2
Regular aspirin user (%)	31	32	32	35
Body mass index (kg/m^2^)‡	24.4	24.5	24.3	24.0
Physical activity, METS/wk (%)§	11.1	13.8	15.8	15.6
Post-menopausal (%)	44	44	44	44
Never used hormones (%)	64	62	61	59
Past use of hormones (%)	18	19	20	22
Current use of hormones (%)	18	19	19	19
Current multivitamin use (%)	8	12	23	84
Prior lower endoscopy (%)	2	2	2	2
Colorectal cancer in a parent or sibling (%)	2	2	2	2
History of prior polyps (%)	8	8	8	8

*Dietary intake and other characteristics at baseline questionnaire in 1980 (mean value, unless otherwise indicated).

Ψ All values have been directly standardized according to the age distribution of the cohort.

† Pack-years were calculated for former and current smokers only.

‡ The body-mass index is the weight in kilograms divided by the square of the height in meters.

§ METS are metabolic equivalents. This was calculated based on the frequency of physical activities (such as jogging) in 1986.

We documented 375 incident cases of colon cancer accessible for quantifying DNA methylation during 1,861,934 person-years. Using DNA methylation assays on these 375 colon cancers, 87 (23%) tumors were found to be CIMP-high. Similarly, *BRAF* mutational status was available for 386 accessible incident colon cancer cases documented during 1,861,927 person years, of which 86 (22%) were *BRAF* mutated.

As in our previous studies [18], [26], [43], [44], we observed an inverse association between folate and vitamin B_6_ intake and colon cancer risk among all cases in our cohort (Tables 1 and 2).

**Table 2:** Risk of colon cancer according to baseline quintiles of one-carbon nutrient intake by CIMP expression in tumors among 88,691 women in the Nurses' Health Study (1980–2002).

Energy-adjusted daily one-carbon nutrient intake	RR (95% CI)*					P Trend
**Folate (µg)**	**Q1**	**Q2**	**Q3**	**Q4**		
	<200	200–299	300–399	≥400		
Cases/Person-years	100/439203	120/606372	55/273313	100/543046		
All cancers‡	1.0	0.78 (0.60–1.02)	0.75 (0.54–1.04)	0.71 (0.53–0.93)		0.06
All cancers*	1.0	0.81 (0.62–1.06)	0.80 (0.57–1.12)	0.78 (0.59–1.05)		0.23
Cases/Person-years	80/439218	92/606395	41/273323	75/543075		
CIMP-low/0‡	1.0	0.75 (0.55–1.01)	0.70 (0.48–1.01)	0.66 (0.48–0.91)		0.04
CIMP-low/0*	1.0	0.78 (0.58–1.05)	0.75 (0.51–1.10)	0.73 (0.53–1.02)		0.15
Cases/Person-years	20/439270	28/606450	14/273348	25/534107		
CIMP-high‡	1.0	0.91 (0.51–1.61)	0.95 (0.48–1.88)	0.88 (0.49–1.59)		0.82
CIMP-high*	1.0	0.95 (0.53–1.68)	1.02 (0.51–2.02)	0.98 (0.54–1.77)		0.94
**Methionine (g)**	**Q1**	**Q2**	**Q3**	**Q4**	**Q5**	
	≤1.50	1.51–1.70	1.71–1.90	1.91–2.20	≥2.21	
Cases/Person-years	78/371363	87/371303	63/375394	62/372422	85/371052	
All cancers‡	1.0	1.1 (0.81–1.49)	0.77 (0.55–1.07)	0.75 (0.54–1.04)	0.96 (0.71–1.31)	0.81
All cancers*	1.0	1.09 (0.80–1.49)	0.76 (0.54–1.06)	0.74 (0.53–1.04)	0.96 (0.70–1.32)	0.87
Cases/Person-years	56/371384	76/371713	51/375402	39/372445	66/371068	
CIMP-low/0‡	1.0	0.90 (0.64–1.27)	1.03 (0.70–1.51)	0.74 (0.49–1.11)	1.00 (0.69–1.43)	0.44
CIMP-low/0*	1.0	0.75 (0.53–1.07)	0.85 (0.58–1.25)	0.65 (0.43–0.98)	1.04 (0.73–1.49)	0.48
Cases/Person-years	22/371411	11/371771	12/375349	23/372449	19/371104	
CIMP-high‡	1.0	0.39 (0.19–0.81)	0.56 (0.28–1.13)	1.06 (0.59–1.91)	0.69 (0.37–1.27)	0.37
CIMP-high*	1.0	0.49 (0.24–1.01)	0.51 (0.25–1.04)	0.97 (0.54–1.75)	0.77 (0.41–1.42)	0.35
**Vitamin B6 (mg)**	**Q1**	**Q2**	**Q3**	**Q4**	**Q5**	
	≤1.30	1.31–1.60	1.61–2.00	2.01–3.50	≥3.51	
Cases/Person-years	89/375530	75/372933	70/3744309	64/371267	21/367894	
All cancers‡	1.0	0.78 (0.58–1.06)	0.66 (0.48–0.98)	0.63 (0.46–0.87)	0.74 (0.54–1.00)	0.48
All cancers*	1.0	0.80 (0.58–1.11)	0.70 (0.48–1.01)	0.70 (0.45–1.10)	0.86 (0.56–1.33)	0.22
Cases/Person-years	72/375549	56/372946	55/374322	49/371278	56/367915	
CIMP-low/0‡	1.0	1.35 (0.98–1.96)	0.65 (0.45–0.92)	0.59 (0.41–0.86)	0.66 (0.46–0.94)	0.12
CIMP-low/0*	1.0	1.36 (0.94–1.96)	0.68 (0.45–1.01)	0.66 (0.41–1.07)	0.77 (0.48–1.23)	0.43
Cases/Person-years	17/375591	19/372973	15/374359	15/371307	21/367944	
CIMP-high‡	1.0	1.04 (0.54–1.99)	0.74 (0.37–1.49)	0.77 (0.38–1.54)	1.05 (0.55–1.99)	0.57
CIMP-high*	1.0	1.06 (0.55–2.06)	0.79 (0.38–1.62)	0.86 (0.40–1.85)	1.24 (0.61–2.52)	0.31
**Vitamin B12 (g)**	**Q1**	**Q2**	**Q3**	**Q4**	**Q5**	
	≤4.0	4.1–5.0	5.1–7.0	7.1–11.0	≥11.1	
Cases/Person-years	98/481642	60/293090	75/317891	64/391812	78/377499	
All cancers‡	1.0	0.95 (0.69–1.31)	1.08 (0.80–1.46)	0.74 (0.54–1.01)	0.89 (0.66–1.20)	0.77
All cancers*	1.0	0.94 (0.68–1.30)	1.08 (0.80–1.47)	0.76 (0.55–1.05)	0.94 (0.69–1.28)	0.94
Cases/Person-years	75/481663	48/293100	55/317904	47/391828	63/377516	
CIMP-low/0‡	1.0	1.01 (0.70–1.45)	1.04 (0.73–1.47)	0.71 (0.49–1.02)	0.94 (0.67–1.31)	0.79
CIMP-low/0*	1.0	1.01 (0.71–1.46)	1.04 (0.73–1.47)	0.73 (0.50–1.05)	0.99 (0.70–1.39)	0.93
Cases/Person-years	23/481706	12/293130	20/317936	17/391850	15/377553	
CIMP-high‡	1.0	0.81 (0.40–1.62)	1.23 (0.68–2.24)	0.83 (0.44–1.56)	0.73 (0.38–1.40)	0.91
CIMP-high*	1.0	0.80 (0.40–1.62)	1.23 (0.68–2.25)	0.86 (0.46–1.62)	0.77 (0.40–1.49)	1.00
**Alcohol (g)**	**Q1**	**Q2**	**Q3**	**Q4**		
	No alcohol	<5 g/day	5–14.9 g/day	≥15 g/day		
Cases/Person-years	111/595227	139/629283	77/416138	48/221285		
All cancers‡	1.0	1.25 (0.98–1.61)	0.99 (0.74–1.33)	1.12 (0.80–1.57)		0.88
All cancers*	1.0	1.27 (0.99–1.63)	1.01 (0.75–1.36)	1.11 (0.78–1.58)		0.98
Cases/Person-years	80/595254	113/629305	59/416154	36/221297		
CIMP-low/0‡	1.0	0.71 (0.53–0.94)	1.06 (0.76–1.48)	1.16 (0.79–1.72)		0.91
CIMP-low/0*	1.0	0.70 (0.52–0.93)	1.08 (0.77–1.52)	1.16 (0.77–1.73)		0.79
Cases/Person-years	31/595293	26/629378	18/416189	12/221315		
CIMP-high‡	1.0	0.84 (0.50–1.41)	0.83 (0.46–1.48)	1.00 (0.51–1.95)		0.60
CIMP-high*	1.0	0.85 (0.50–1.43)	0.84 (0.47–1.51)	1.00 (0.51–1.95)		0.67

‡ Age adjusted only.

*All models are adjusted for age (continuous), energy intake, gender, screening sigmoidoscopy, family history of colorectal cancer, aspirin use, smoking, physical activity in METs, baseline body mass index, a history of colon polyps, beef intake, calcium, multi-vitamin use, and baseline folate, vitamin B6, B12, methionine, and alcohol if not primary exposure. P for heterogeneity of the association for folate intake and CIMP-low/0 colon cancer versus folate intake and CIMP-high colon cancers = 0.73 (χ^2^ = 1.31, 3 d.f.).

P for heterogeneity of the association for vitamin B_6_ intake and CIMP-high colon cancer and vitamin B_6_ intake and CIMP-low/0 colon cancers = 0.63 (χ^2^ = 2.6, 4 d.f.) and for vitamin B_12_ intake = 0.94 (χ^2^ = 0.79, 4 d.f.). P for heterogeneity of the association for methionine intake and CIMP-high colon cancer and methionine intake and CIMP-low/0 colon cancers = 0.007 (χ^2^ = 14.097, 4 d.f.), and for alcohol intake = 0.32 (χ^2^ = 3.52, 3 d.f.).

We further evaluated the influence of one-carbon nutrients on colon cancer risk according to CIMP status in colon cancer. Overall, none of the associations examined reached statistical significance at the conservative significance level (p = 0.01). For total folate intake (Table 2), the benefit of consumption beyond 200 µg per day appeared to be restricted to CIMP-low/0 cancers. Compared to women with less than 200 µg folate per day, the multivariate RR for the development of CIMP-low/0 colon cancer among those reporting 400 µg or more of folate intake per day was 0.73 (95% CI, 0.53 to 1.02; P for trend = 0.15) though statistical significance was not reached. In contrast, total folate intake had no influence on the risk of CIMP-high tumors (RR, 0.98; 95% CI, 0.54–1.77).

We further examined the influence of vitamin B_6_ and B_12_ intake according to CIMP status (Table 2). The benefit of vitamin B_6_ intake also appeared confined to CIMP-low/0 cancer (multivariate RR 0.77; 95% CI, 0.48 to 1.23 comparing extreme quintiles), whereas B_6_ intake had no influence on the risk of CIMP-high tumors. However, there was no differential effect of vitamin B_12_ on colon cancer risk by tumoral CIMP status and none of the observed associations reached statistical significance.

We also examined the influence of methionine intake and alcohol intake according to CIMP status (Table 2). There appeared to be a modestly greater reduction in the risk of CIMP-high cancers with increasing methionine intake. We did not observe a significant influence of alcohol intake on colon cancer risk, and the effect did not appear to differ according to CIMP status.

When using our 16-marker panel, overall, results remained unchanged (data not shown).

Similarly, for *BRAF* mutation status, none of the examined nutrients displayed a significant effect overall, or by mutational status (Table 3).

**Table 3:** Risk of colon cancer according to baseline quintiles of one-carbon nutrient intake by BRAF mutation status in tumors among 88,691 women in the Nurses' Health Study (1980–2002).

Energy-adjusted daily one-carbon nutrient intake	RR (95% CI)*					P Trend
**Folate (µg)**	**Q1**	**Q2**	**Q3**	**Q4**	**Q5**	
	<200	200–299	300–399	≥400		
Cases/Person-years	102/439203	124/606368	55/273313	105/543043		
All cancers‡	1.0	0.79 (0.61–1.03)	0.73 (0.53–1.02)	0.73 (0.55–0.95)		0.10
All cancers*	1.0	0.83 (0.63–1.08)	0.79 (0.57–1.11)	0.81 (0.61–1.08)		0.37
Cases/Person-years	79/439222	99/606387	43/273321	79/543064		
*BRAF* mutation (−)‡	1.0	0.70 (0.40–1.24)	0.71 (0.35–1.42)	0.80 (0.45–1.39)		0.86
*BRAF* mutation (−)*	1.0	0.74 (0.42–1.30)	0.76 (0.38–1.54)	0.89 (0.51–1.57)		0.87
Cases/Person-years	23/439265	25/606455	12/273350	26/543113		
*BRAF* mutation (+)‡	1.0	0.81 (0.60–1.09)	0.74 (0.51–1.07)	0.71 (0.52–0.96)		0.08
*BRAF* mutation (+)*	1.0	0.85 (0.63–1.15)	0.80 (0.55–1.17)	0.80 (0.57–1.09)		0.26
**Methionine (g)**	**Q1**	**Q2**	**Q3**	**Q4**	**Q5**	
	≤1.50	1.51–1.70	1.71–1.90	1.91–2.20	≥2.21	
Cases/Person-years	82/371361	90/371701	65/375393	63/372420	86/371052	
All cancers‡	1.0	1.08 (0.80–1.46)	0.76 (0.55–1.05)	0.72 (0.52–1.00)	0.92 (0.68–1.25)	0.62
All cancers*	1.0	1.07 (0.79–1.45)	0.74 (0.53–1.02)	0.70 (0.50–0.98)	0.91 (0.66–1.24)	0.61
Cases/Person-years	24/371412	13/371772	12/375440	19/372454	18/371107	
*BRAF* mutation (−)‡	1.0	1.87 (0.95–3.68)	0.48 (0.24–0.95)	0.74 (0.41–1.36)	0.66 (0.36–1.22)	0.88
*BRAF* mutation (−)*	1.0	1.90 (0.96–3.73)	0.46 (0.23–0.93)	0.73 (0.40–1.33)	0.65 (0.35–1.20)	0.87
Cases/Person-years	58/371382	77/371710	53/375400	44/372438	68/371064	
*BRAF* mutation (+)‡	1.0	1.31 (0.93–1.84)	0.87 (0.60–1.26)	0.71 (0.48–1.06)	1.03 (0.73–1.47)	0.64
*BRAF* mutation (+)*	1.0	1.29 (0.92–1.82)	0.85 (0.58–1.24)	0.70 (0.47–1.03)	1.01 (0.71–1.45)	0.62
**Vitamin B6 (mg)**	**Q1**	**Q2**	**Q3**	**Q4**	**Q5**	
	≤1.30	1.31–1.60	1.61–2.00	2.01–3.50	≥3.51	
Cases/Person-years	93/375527	77/372932	70/374309	66/371265	80/367894	
All cancers‡	1.0	0.77 (0.57–1.04)	0.63 (0.46–0.87)	0.62 (0.45–0.85)	0.73 (0.54–0.99)	0.85
All cancers*	1.0	0.77 (0.56–1.07)	0.66 (0.46–0.94)	0.66 (0.42–1.03)	0.82 (0.53–1.26)	0.52
Cases/Person-years	19/375591	15/372978	16/374359	13/371309	23/367948	
*BRAF* mutation (−)‡	1.0	1.37 (0.70–2.69)	0.71 (0.36–1.37)	0.60 (0.29–1.21)	1.03 (0.56–1.89)	0.28
*BRAF* mutation (−)*	1.0	1.36 (0.68–2.69)	0.73 (0.37–1.47)	0.64 (0.30–1.38)	1.15 (0.58–2.28)	0.19
Cases/Person-years	74/375546	62/372940	54/374323	53/371275	57/367911	
*BRAF* mutation (+)‡	1.0	0.78 (0.56–1.09)	0.62 (0.43–0.87)	0.62 (0.44–0.89)	0.66 (0.46–0.93)	0.62
*BRAF* mutation (+)*	1.0	0.78 (0.55–1.12)	0.64 (0.43–0.95)	0.67 (0.42–1.07)	0.73 (0.46–1.16)	0.89
**Vitamin B12 (g)**	**Q1**	**Q2**	**Q3**	**Q4**	**Q5**	
	≤4.0	4.1–5.0	5.1–7.0	7.1–11.0	≥11.1	
Cases/Person-years	104/481636	61/293088	75/317892	68/391811	78/377500	
All cancers‡	1.0	0.91 (0.66–1.24)	1.02 (0.76–1.37)	0.74 (0.54–1.00)	0.84 (0.62–1.12)	0.57
All cancers*	1.0	0.90 (0.66–1.24)	1.02 (0.75–1.37)	0.76 (0.56–1.03)	0.88 (0.65–1.19)	0.72
Cases/Person-years	24/481709	11/293131	18/317938	17/391847	16/377559	
*BRAF* mutation (−)‡	1.0	1.41 (0.69–2.89)	1.06 (0.58–1.95)	0.80 (0.43–1.48)	0.74 (0.40–1.40)	0.95
*BRAF* mutation (−)*	1.0	1.42 (0.70–2.91)	1.06 (0.57–1.95)	0.82 (0.44–1.53)	0.78 (0.42–1.48)	0.86
Cases/Person-years	80/481653	50/293098	57/317903	51/391829	62/377510	
*BRAF* mutation (+)‡	1.0	0.97 (0.68–1.38)	1.01 (0.72–1.42)	0.72 (0.51–1.02)	0.86 (0.62–1.21)	0.46
*BRAF* mutation (+)*	1.0	0.96 (0.67–1.37)	1.00 (0.71–1.41)	0.74 (0.52–1.05)	0.92 (0.65–1.28)	0.60
**Alcohol (g)**	**Q1**	**Q2**	**Q3**	**Q4**		
	No alcohol	<5 g/day	5–14.9 g/day	≥15 g/day		
Cases/Person-years	112/595229	145/629277	81/416136	48/221285		
All cancers‡	1.0	1.29 (1.01–1.66)	1.04 (0.78–1.38)	1.11 (0.79–1.55)		0.98
All cancers*	1.0	1.32 (1.03–1.70)	1.07 (0.80–1.43)	1.11 (0.79–1.58)		0.87
Cases/Person-years	23/595301	33/629373	18/416192	12/221319		
*BRAF* mutation (−)‡	1.0	0.38 (0.24–0.59)	1.21 (0.61–2.08)	1.35 (0.67–2.71)		0.75
*BRAF* mutation (−)*	1.0	0.68 (0.40–1.17)	1.16 (0.62–2.15)	1.36 (0.67–2.74)		0.80
Cases/Person-years	89/595247	112/629303	63/416150	36/221294		
*BRAF* mutation (+)‡	1.0	1.84 (1.42–2.38)	1.48 (1.09–2.00)	1.52 (1.05–2.21)		0.84
*BRAF* mutation (+)*	1.0	1.29 (0.97–1.70)	1.05 (0.75–1.46)	1.05 (0.71–1.56)		0.75

‡ Age adjusted only.

*All models are adjusted for age (continuous), energy intake, gender, screening sigmoidoscopy, family history of colorectal cancer, aspirin use, smoking, physical activity in METs, baseline body mass index, a history of colon polyps, beef intake, calcium, multi-vitamin use, and baseline folate, vitamin B_6_, B_12_, methionine, and alcohol if not primary exposure.

## Discussion

Our current study is a prototypical study in “*Molecular Pathological Epidemiology*”, the concept of which has been recently described and consolidated by Ogino et al. [45]. It represents a relatively new, specialized field of epidemiology based on the molecular classification of cancer. In molecular pathologic epidemiology, a known or suspected etiologic factor is examined in relation to a specific somatic molecular change, in order to gain insights into the carcinogenic mechanism [45]. Assume, a given etiologic factor (lifestyle, dietary, environmental or genetic) is hypothesized to cause a certain somatic molecular change. If we can demonstrate a specific relationship between the etiologic factor and the molecular change, it can provide evidence for causality. In addition, for an individual who has a susceptibility to a specific somatic molecular change leading to cancer, we may be able to develop a personalized preventive measure, which targets specific molecules or pathways [45]. Therefore, molecular pathologic epidemiology research can contribute to deciphering the carcinogenic process as well as optimizing preventive strategies [45].

In this prospective cohort study, we found suggestions for both low folate and vitamin B_6_ intakes to be associated with an increased risk of CIMP-low/0 colon cancers but not with that of CIMP-high tumors. The elevation in risk was principally limited to participants with the lowest levels of folate and vitamin B_6_ intake. In addition, we observed that the effect of a higher methionine intake on colon cancer risk modestly differed by tumoral CIMP status, with the beneficial effect of methionine being principally limited to CIMP-high cancer. There was no difference in the effects of folate or B vitamins on colon cancers stratified by *BRAF* status. Moreover, none of the observed associations reached statistical significance.

Mechanistically, it appears plausible that chronic folate deficiency may be involved in CpG island methylation [46]. In a Japanese population, reduced activity of the enzyme methylenetetrahydrofolate reductase (MTHFR) due to the *MTHFR* codon 429 variant (rs1801131), thereby impairing folate metabolism, has been associated with CIMP-high tumors in the proximal colon [47]. In a Caucasian population, genetic variation in the *MTHFR* rs1801131 variant appears to increase risk of CIMP-high colon tumors [48], [49] although this has not been confirmed by the Netherlands Cohort Study data [22], [50]. The relation between the *MTHFR* SNP and CIMP in colon cancer is strong in combination with low folate and methionine intake as well as high alcohol consumption [49], [51]. A more recent study suggests that low expression of gamma-glutamyl hydrolase (GGH) is strongly associated with CIMP-high tumors, providing further support for a role of one-carbon metabolism in this phenotype [52].

A prior case-control study of 1,154 colon tumors assessed the influence of one-carbon nutrient intake on CIMP as well as *BRAF* status in these tumors and found them not to be associated with either CIMP-high or *BRAF* mutation [51]. However, compared to our current study, nonquantitative methylation-specific PCR on a different CpG island panel, and a less stringent definition of CIMP-high (2 or more of 5 markers methylated) were used in that study. Interestingly, the authors also observed that obesity was associated with a two-fold risk of having a non-CIMP-high tumor [51]. In our study, after carefully adjusting for obesity, both folate and vitamin B_6_ (the intake of which is highly correlated in our population) were associated with risk of CIMP-low/0 tumors.

In another case-case study within the Netherlands Cohort Study, colorectal cancer patients in the low folate/high alcohol intake group are more likely to have promoter hypermethylation than patients in the high folate/low alcohol group [53]. Notably, the Netherlands Cohort Study has shown a possible association between vitamin B_6_ and *MLH1* methylation [22] and an inverse association between *MTR* rs1805087 polymorphism and CIMP in men [22].

In one of the most recent study to evaluate associations between one-carbon metabolism and colon cancer by CIMP/*BRAF* status, to date, utilizing a nested case-control approach within the Northern Sweden Health and Disease study (190 cases and 1∶2 matched controls) [24], van Guelpen et al. show a lower risk of CIMP-low/CIMP-high colorectal tumors with very low folate levels. Collectively, there is accumulating evidence for the hypothesis that a balance between various metabolic intermediates of methyl-group influences locus-specific CpG island methylation reaction. Utilizing data from an adenoma prevention trial of folic acid and aspirin, finally, higher RBC folate levels, but not higher dietary folate consumption, was associated with higher methylation levels [21].

Besides influence of one-carbon nutrients, evidence suggests that local DNA sequence context may influence assembly of a methylation reaction machinery and locus-specific DNA methylation. Studies have shown that *cis*-acting elements cause allele-specific methylation in the mammalian genome [54], [55], [56]. Thus, germline variations in *cis*-acting elements may influence epigenetic status including DNA methylation. The *MLH1* rs1800734 promoter SNP has been associated with *MLH1* promoter methylation [57], [58] and MSI [59] in colorectal cancer. Another study has shown that *MGMT* rs16906252 promoter SNP is strongly associated with *MGMT* promoter methylation and loss of expression in colorectal cancer [60], and with *MGMT* methylation in peripheral blood cells and normal colonic mucosa in individuals without colorectal cancer [61], [62]. It remains to be investigated whether these or other *cis*-acting germline variants interact with other one-carbon-related factors to modify susceptibility to aberrant DNA methylation.

Our study has several important strengths. First, because we collected detailed, updated information on a number of dietary and lifestyle covariates relevant to colon carcinogenesis over 22 years of follow-up and with high follow-up rates, we were able to examine long-term exposures to one-carbon nutrients and to take into consideration important confounding factors. Second, our study is prospective, eliminating concerns on differential recall bias, particularly with regard to our dietary assessments. Any remaining bias from exposure misclassification were thus likely nondifferential by nature, biasing our results only toward the null. Further, we have successfully linked these nutrients—as assessed via a semiquantitative food frequency questionnaire (SFFQ)—to other relevant endpoints in prior analyses, indicating that measurement error is not large enough to hide any real associations.

Limitations of note relate to folate fortification, which became mandatory in 1998 [63]. We have multiple assessments of one-carbon nutrient intakes prior to fortification. Further, our results remained unchanged when restricted to cases that arose prior to folate fortification (1998), which has also been demonstrated in prior analyses [20], [42], [64]. Other limitations and caveats specific to molecular pathologic epidemiology in general have been discussed elsewhere [45].

We cannot exclude the possibility of residual confounding as a potential explanation for our findings; nonetheless, in our multivariate analyses which included many known or suspected risk factors for colon cancer, the multivariate risk estimates did not materially differ from the age-adjusted results. Further, we were unable to obtain tumor tissue from all cases of confirmed colon cancer detected in the Nurses' Health Study cohort, but the risk factors in these cases did not appreciably differ from those in cases with tumor tissue available. Finally, even prior to mandated fortification in 1998 [63], our participants still had relatively high folate and vitamin B_6_ levels. It is therefore possible that we might have found even stronger associations in the present study if our folate levels would have included an even lower range.

In summary, we demonstrate that the reduced risk of colon cancer associated with replete folate status is somewhat limited to CIMP-low/0 cancers. Additional studies are necessary to elucidate the exact mechanism of an abnormality in one-carbon metabolism leading to aberrant CpG island methylation.
